# Supplementary material for: scDisInFact: disentangled learning for integration and prediction of multi-batch multi-condition single-cell RNA-sequencing data
Source: Nat Commun. 2024 Jan 30;15:912. doi: 10.1038/s41467-024-45227-w (PMC10827746; doi:10.1038/s41467-024-45227-w)
Supplement: Supplementary file 1 — Supplementary Information [file 41467_2024_45227_MOESM1_ESM.pdf]

Supplementary Information

Supplementary Figures and tables

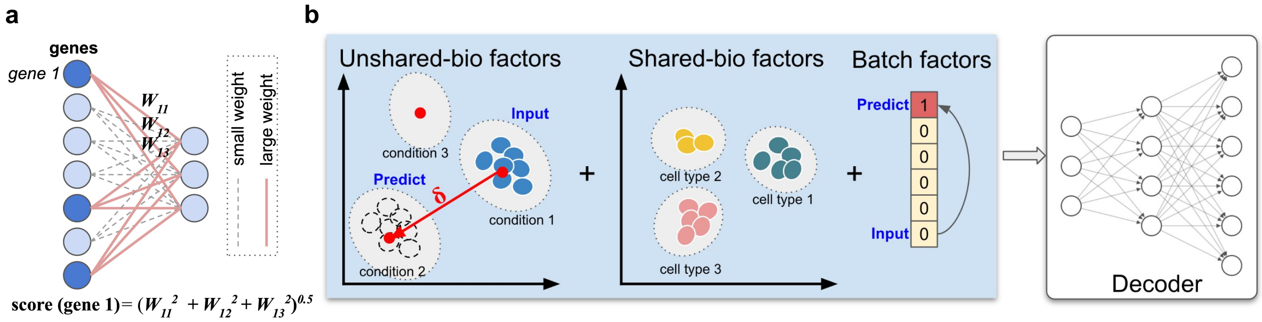

Supplementary Figure 1. The key gene detection and perturbation prediction procedures in scDisInFact. **a**. The first layer of the unshared encoder is designed to extract CKGs. For each gene (e.g. gene 1), a score can be calculated using the weight connected to the gene (e.g. $\sqrt{W_{11}^2 + W_{12}^2 + W_{13}^2}$ for gene 1). **b**. scDisInFact allows for the perturbation prediction across conditions and batches. Given an input count matrix, scDisInFact updates its unshared-bio factors (from the input condition to the predicted condition) and batch factors (from the input batch to the predicted batch), and then feed the updated factors into the decoder to generate the predicted counts. Neural network illustration adapted from LeNail, (2019). NN-SVG: Publication-Ready Neural Network Architecture Schematics. Journal of Open Source Software, 4(33), 747. https://doi.org/10.21105/joss.00747

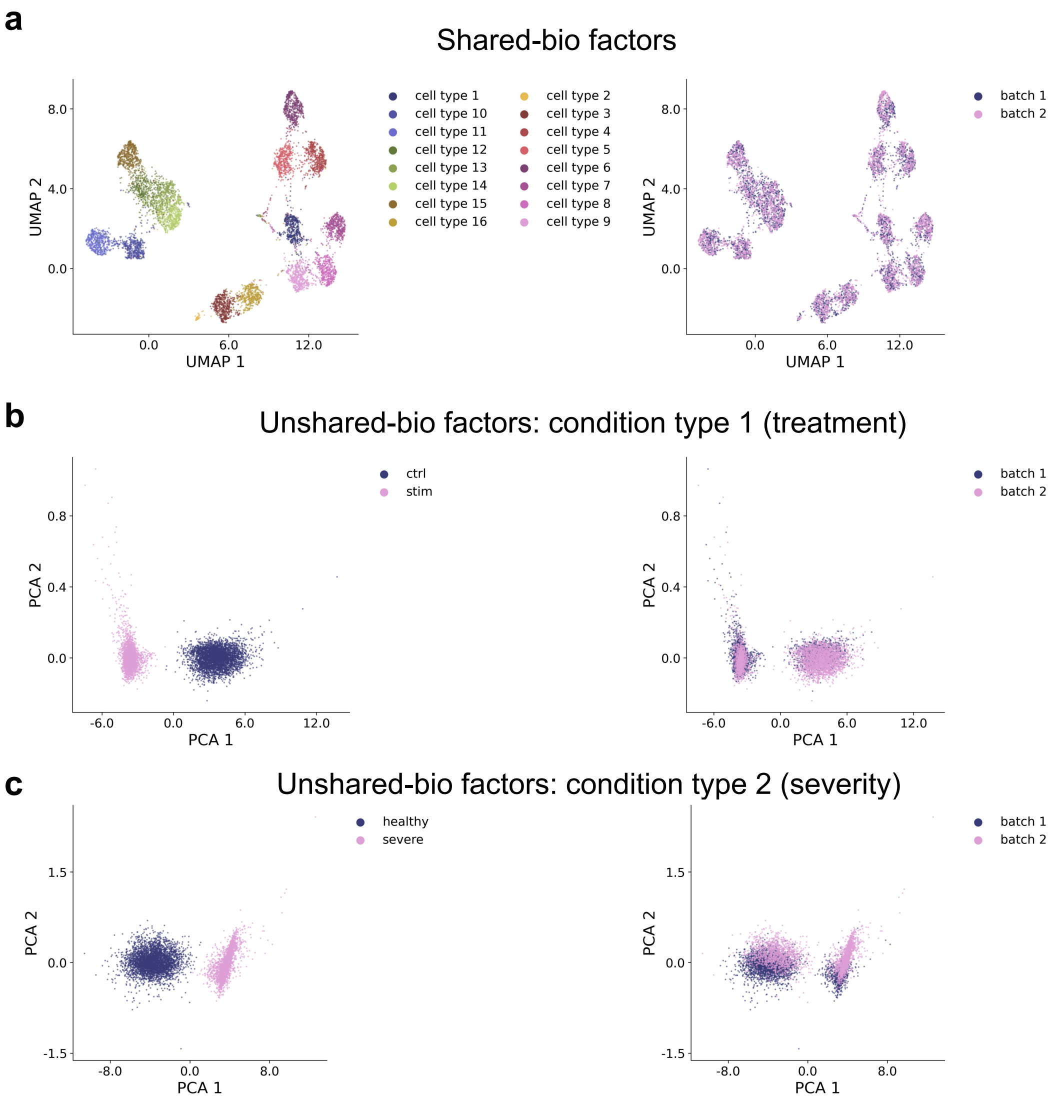

Supplementary Figure 2. Visualization of latent factors learned by scDisInFact on one simulated dataset. **a**. The UMAP visualization of shared-bio factors, where cells are colored by cell type identity (left) and batches (right). **b**. The PCA visualization of unshared-bio factors corresponds to the first condition type, where cells are colored by conditions (left) and batches (right). **c**. The PCA visualization of unshared-bio factors corresponds to the second condition type, where cells are colored by conditions (left) and batches (right). Source data for a, b, and c are provided in the Source Data file.

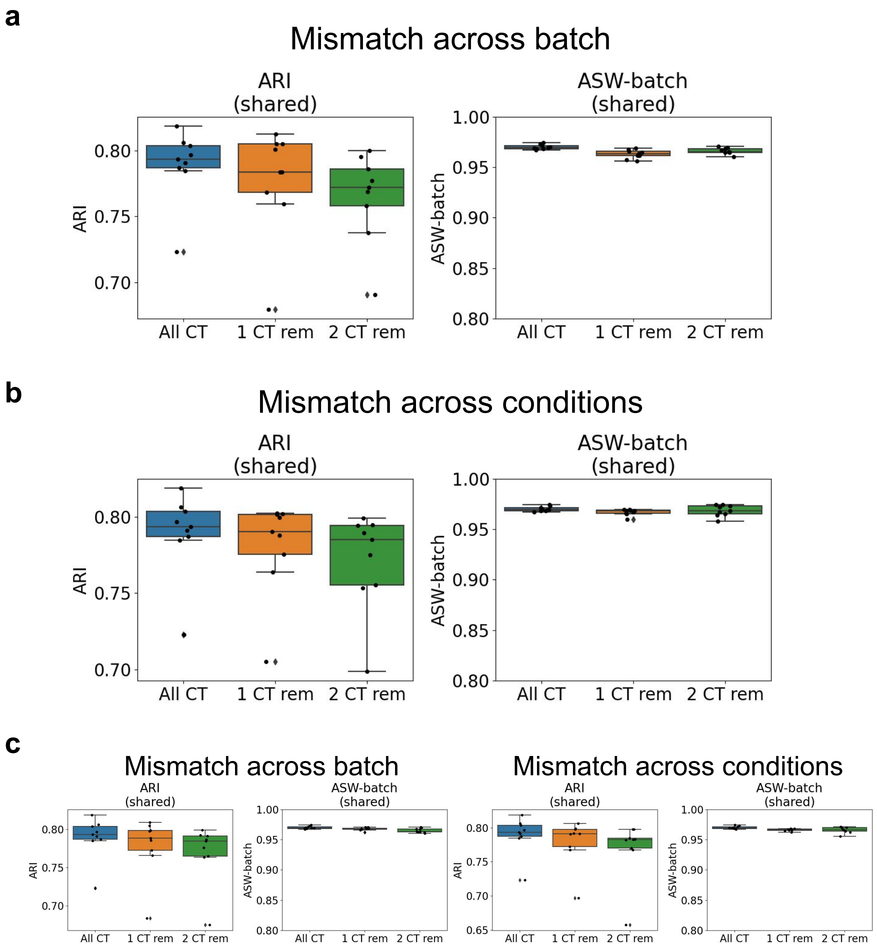

Supplementary Figure 3. The scores of shared-bio factors on datasets with different degrees of cell type mismatch across conditions and batches. **a**. The ARI (left) and ASW-batch (right) scores of shared-bio factors when there are different degrees of cell type mismatch across batches. **b**. The ARI (left) and ASW-batch (right) scores of shared-bio factors when there are different degrees of cell type mismatch across conditions. In a and b, the x-axis shows three cases corresponding to different degrees of mismatch: (1) all cell types are matched (All CT); (2) 1 cell type is not matched (1 CT rem); (3) 2 cell types are not matched (2 CT rem). In the boxplots, the center lines show the median data value, and the box limits show the lower and upper quartiles (25% and 75%, respectively). The length of the whiskers is within 1.5x interquartile range. Outliers beyond the whiskers are plotted as points. $n = 9$ independent samples are included in each box. Source data for a and b are provided in the Source Data file. **c**. The sores of shared-bio factors under different degrees of mismatch. Different sets of clusters are removed in c compared to a and b (clusters 3 and 4).

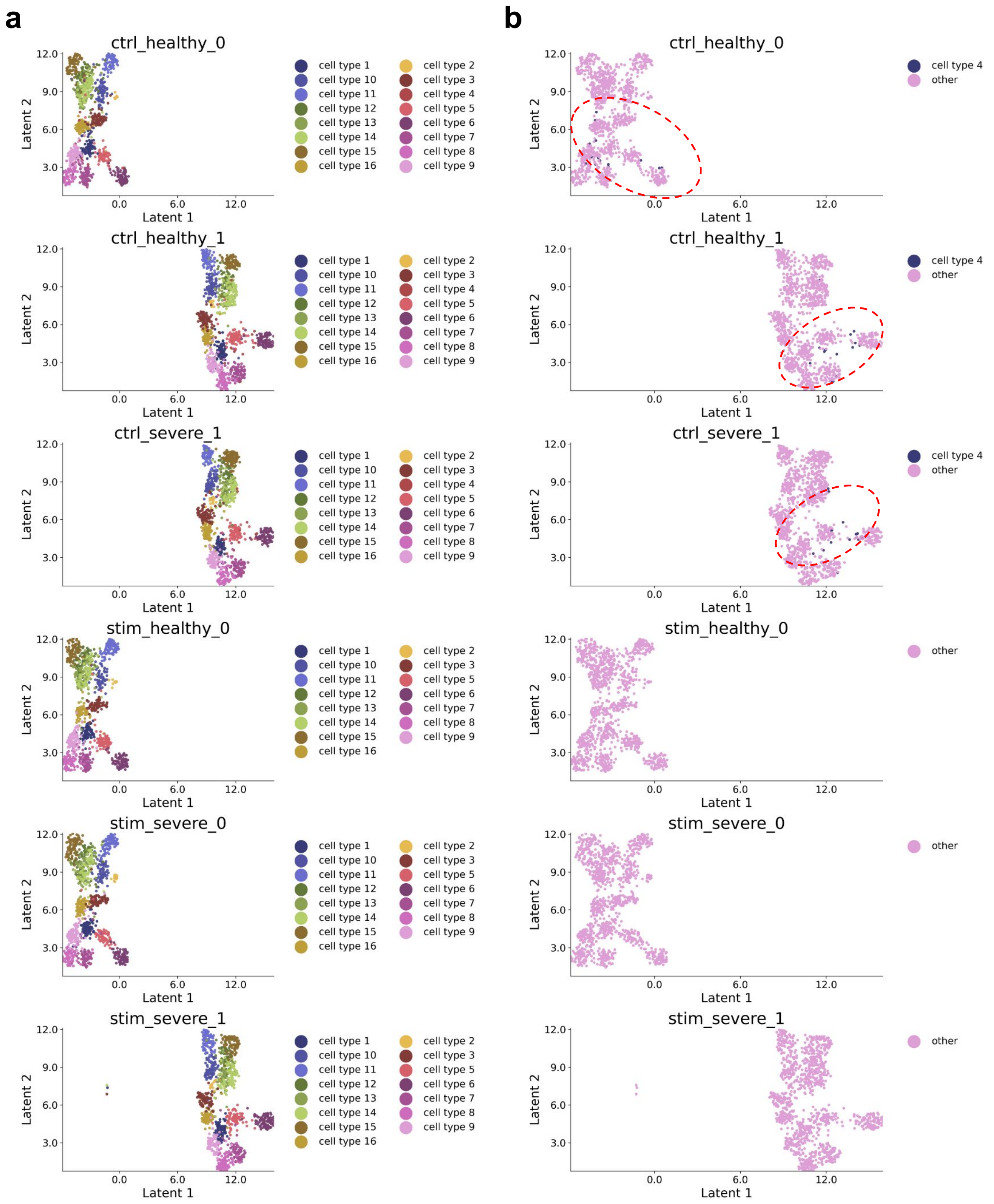

Supplementary Figure 4. The UMAP visualization of the dataset with one rare cell type (cell type 4), where cells are visualized separately according to the batches, and cells are colored according to (**a**) cell type composition and (**b**) rare cell type identity. The cells of rare cell type are scattered across the cell population (red circle). Source data for a and b are provided in the Source Data file.

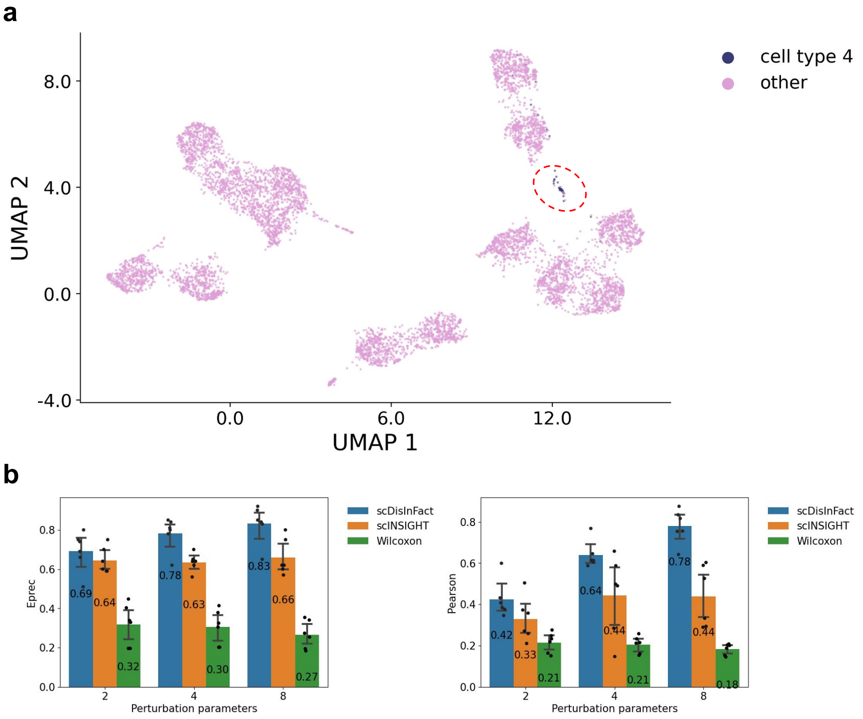

Supplementary Figure 5. Additional test results of scDisInFact on rare cell type separation and CKGs detection. **a**. The UMAP visualization of the shared-bio factors on the dataset with rare cell type (cell type 4). Cells are colored according to the rare cell type identity. **b**. The Early Precision score (Eprec) and Pearson correlation score of scDisInFact in the task of CKGs detection. scDisInFact consistently shows better performance compared to the two baseline methods. In the barplots, $n = 6$ independent samples are included in each bar, and mean is used for each bar. The error bar represents 95% confidence interval, and the center of the error bar shows the mean. Source data for a and b are provided in the Source Data file.

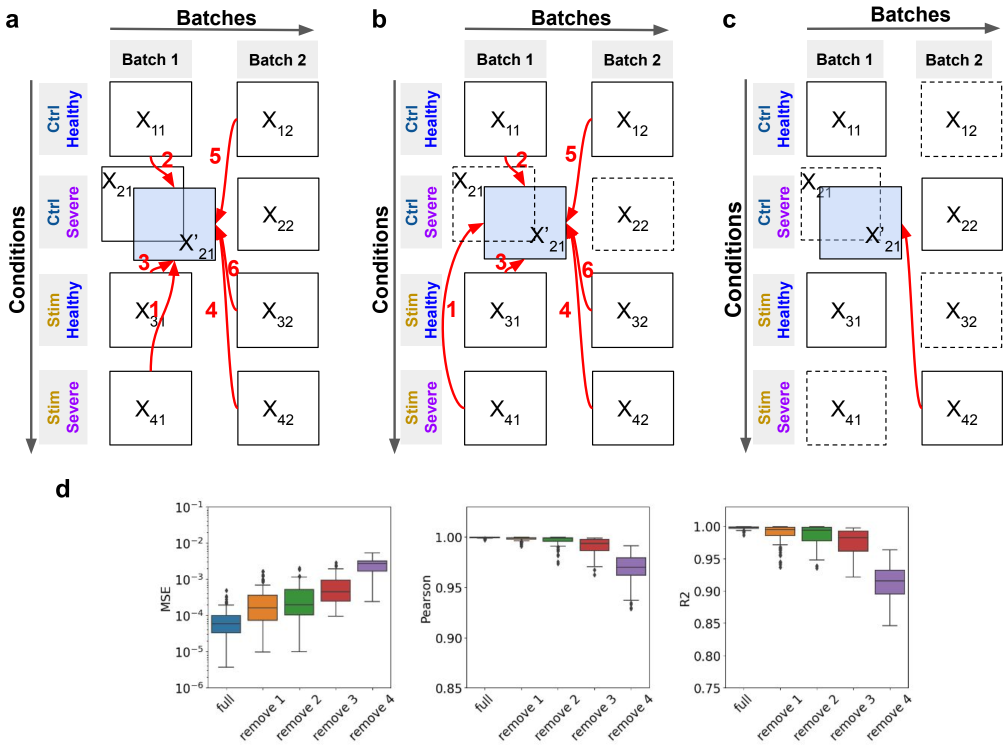

Supplementary Figure 6. Additional perturbation prediction results on simulated datasets. **a**. Graphical illustration of in-sample test, where the red arrows represent the 6 prediction categories. **b**. Graphical illustration of out-of-sample test, where the red arrows represent the 6 prediction categories. **c**. Graphical illustration of prediction with different numbers of held-out matrices. Matrices with dashed borders are the held-out matrices when all 4 matrices are removed. The red arrow shows the direction of perturbation prediction. **d**. Perturbation prediction accuracy of scDisInFact with different numbers of held-out matrices, accuracy is measured with cluster-specific MSE (left), Pearson correlation (middle), and R^2 score (right). In the boxplots, the center lines show the median data value, and the box limits show the lower and upper quartiles (25% and 75%, respectively). The length of the whiskers is within 1.5x interquartile range. Outliers beyond the whiskers are plotted as points. $n = 144$ independent samples are included in each box. Source data for d are provided in the Source Data file.

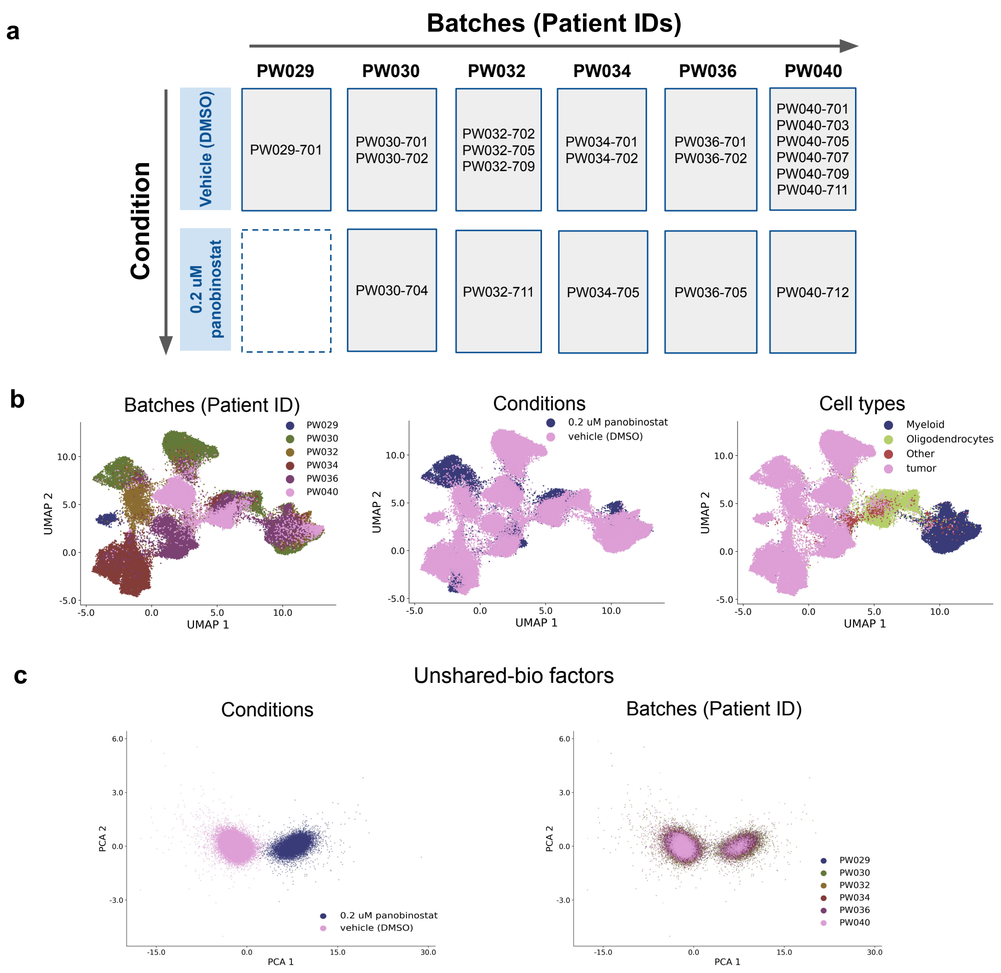

Supplementary Figure 7. Additional test results on GBM dataset. **a**. The arrangement of count matrices in the dataset. The matrices are grouped by conditions (rows) and batches (columns). **b**. The UMAP visualization on the count matrices, where cells are annotated by batches (left), conditions (middle), and cell types (right). Cell-type labels are obtained from the original data paper. **c**. The UMAP visualization of the unshared-bio factors, where cells are annotated by conditions (left) and batches (right). Source data for b and c are provided in the Source Data file.

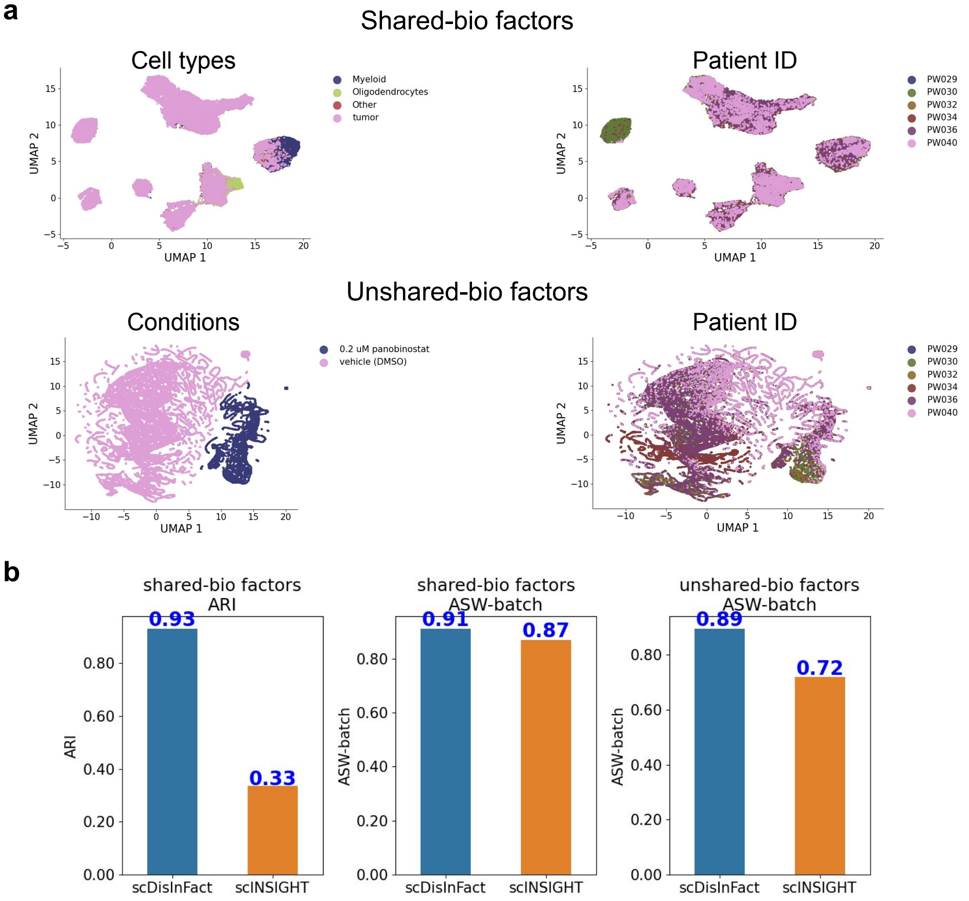

Supplementary Figure 8. Additional test results on GBM dataset. **a**. The visualization of shared-bio (upper) and unshared-bio factors (lower) learned by scINSIGHT. In the visualizations of shared-bio factors, cells are colored by cell types (left) and batches (right). In the visualization of unshared-bio factors, cells are colored by conditions (left) and batches (right). **b**. The disentanglement scores of scDisInFact and scINSIGHT. The metrics include the ARI (left) and ASW-batch (middle) scores for shared-bio factors, and the ASW-batch score (right) for unshared-bio factors. Source data for a and b are provided in the Source Data file.

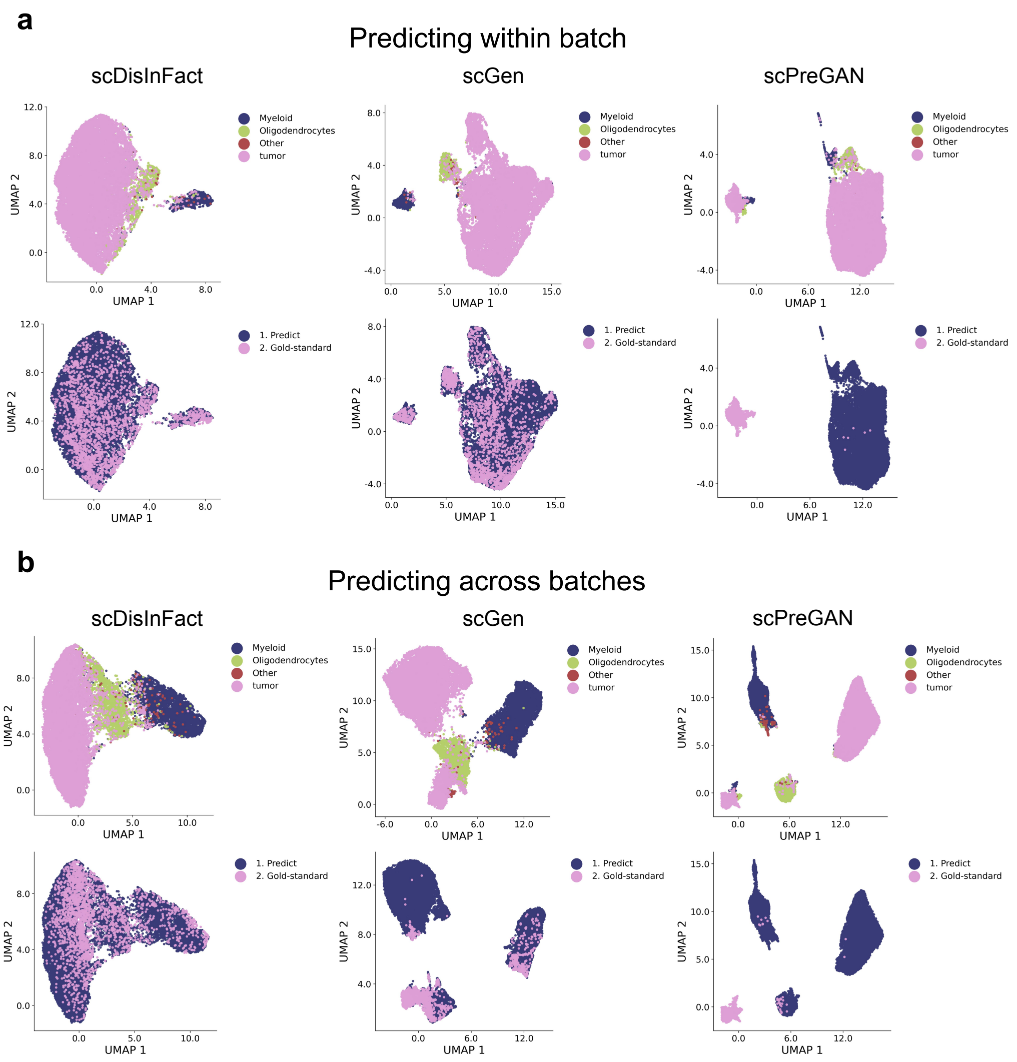

Supplementary Figure 9. UMAP visualization of predicted and gold-standard matrices in the perturbation prediction test of GBM dataset. **a**. The UMAP visualization of the predicted and gold-standard matrices of scDisInFact (left), scGen (middle), and scPreGAN (right) in the prediction task 1 (within batch). **b**. The UMAP visualization of the predicted and gold-standard matrices of scDisInFact (left), scGen (middle), and scPreGAN (right) in the prediction task 2 (across batches). In both (a) and (b), Cells are colored by the original cell type (upper row) and the sources (lower row, predicted or gold-standard). Source data for a and b are provided in the Source Data file.

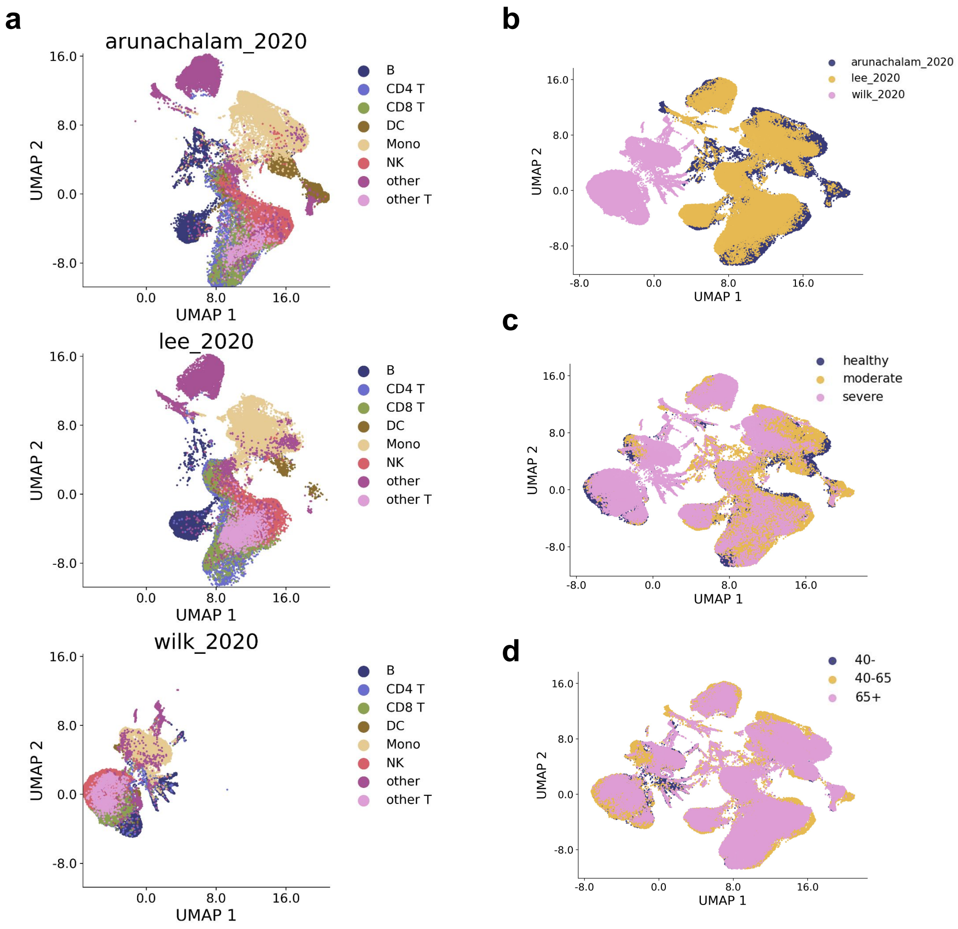

Supplementary Figure 10. UMAP visualization of the count matrices in COVID-19 dataset. **a**. UMAP visualization of cells in different batches ("arunachalam_2020", "lee_2020", and "wilk_2020"), where cells are colored by the input cell types. UMAP was calculated for cells from all three studies together to obtain the (x,y) coordinates for the cells in the UMAP space, but cells from different studies are shown in separate plots. **b-d**. UMAP visualization of cells, where cells are colored by (b) data batches, (c) disease severity, and (d) age groups. Source data for a-d are provided in the Source Data file.

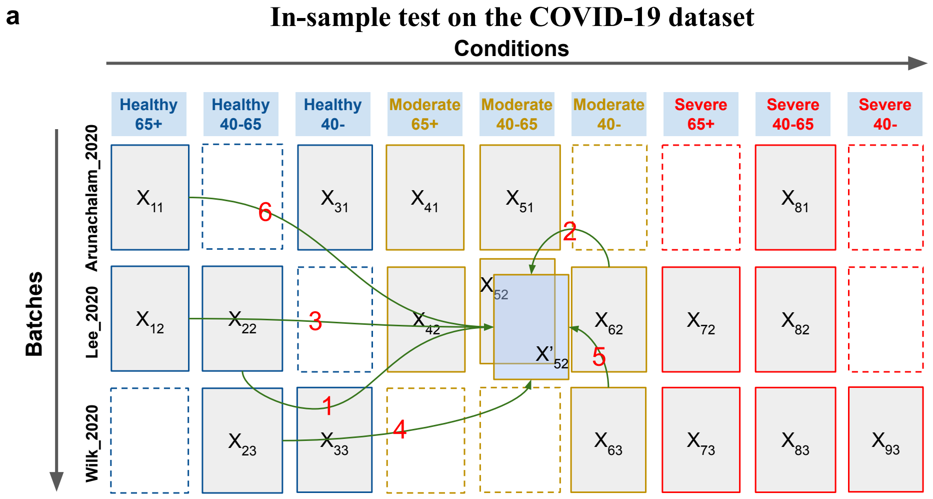

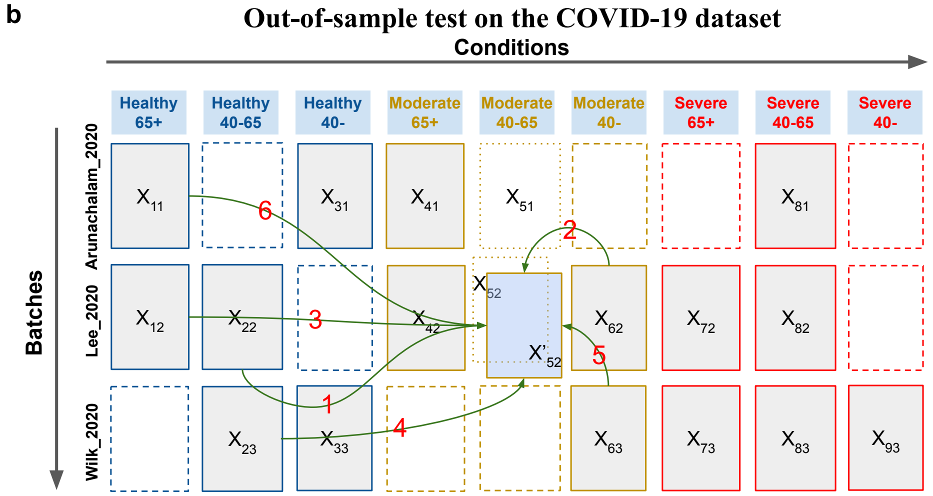

Supplementary Figure 11. Graphical illustration of perturbation prediction test on COVID-19 dataset. **a**. The in-sample perturbation prediction test on COVID-19 dataset. 6 arrows show 6 prediction categories. **b**. The out-of-sample perturbation prediction test on COVID-19 dataset. 6 arrows show 6 prediction categories. Matrices X_{51} and X_{52} are removed in the training data.

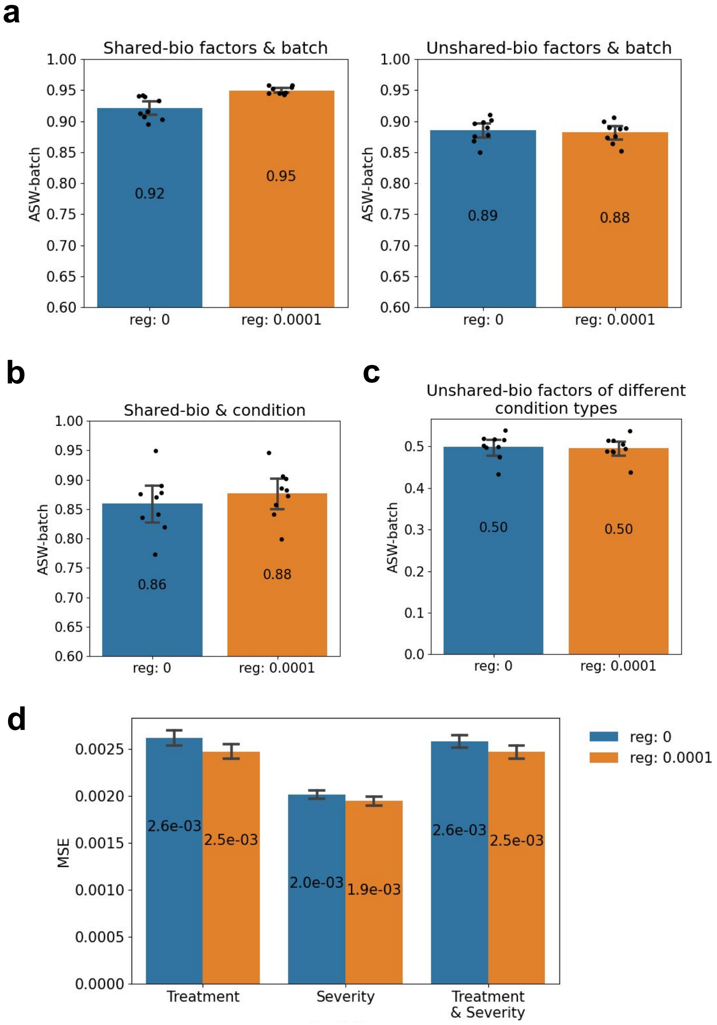

Supplementary Figure 12. Ablation test results of MMD loss. Two scDisInFact models are compared: scDisInFact with and without MMD loss (reg: 0.0001 or reg:0). **a**. The barplot of ASW-batch scores that measures the removal of batch effect in the shared-bio factors and unshared-bio factors. **b**. The barplot of ASW-batch scores that measures the removal of condition effect in the shared-bio factors. **c**. The barplot of ASW-batch scores that measures the disentanglement of unshared-bio factors for different condition types. In the barplots above, $n = 9$ independent samples are included in each bar. The error bar represents 95% confidence interval, and the center of the error bar shows the mean. **d**. The barplot of MSE scores that measures the perturbation prediction accuracy. In all barplots, $n = 11214$ independent samples are included in each bar. The error bar represents 95% confidence interval, and the center of the error bar shows the mean. Source data for a-d are provided in the Source Data file.

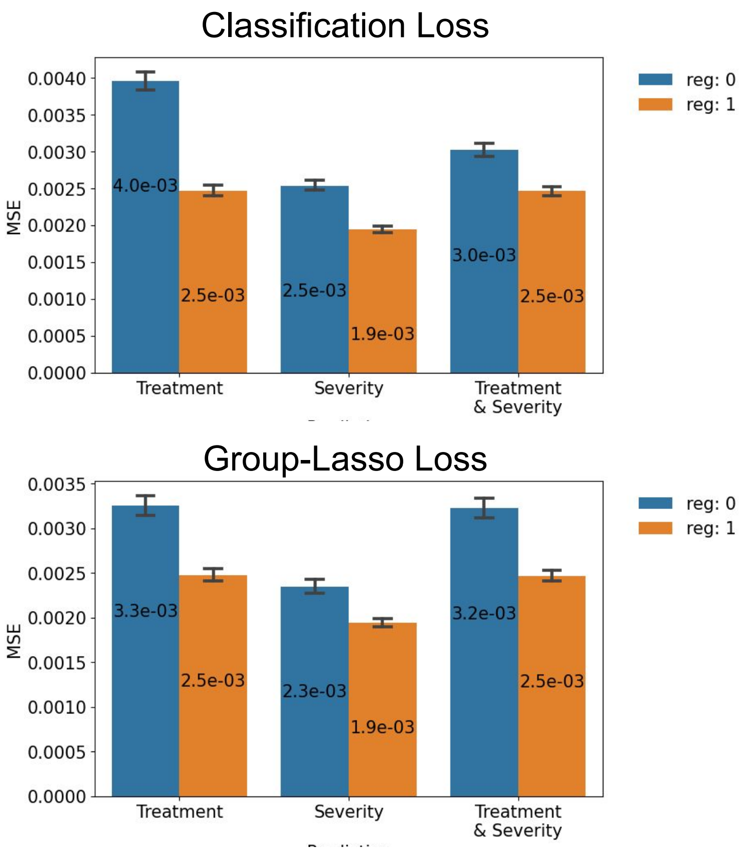

Supplementary Figure 13. Ablation test results of classification loss and group-lasso loss. For the ablation test on classification loss, two models are compared: (1) scDisInFact with classification loss (reg: 1) and scDisInFact without classification loss (reg: 0). For the ablation test on group-lasso loss, two models are compared: (1) scDisInFact with group-lasso loss (reg: 1) and scDisInFact without group-lasso loss (reg: 0) MSE is used to measure the perturbation prediction accuracy of the models. In the barplots, $n = 11214$ independent samples are included in each bar. The error bar represents 95% confidence interval, and the center of the error bar shows the mean. Source data are provided in the Source Data file.

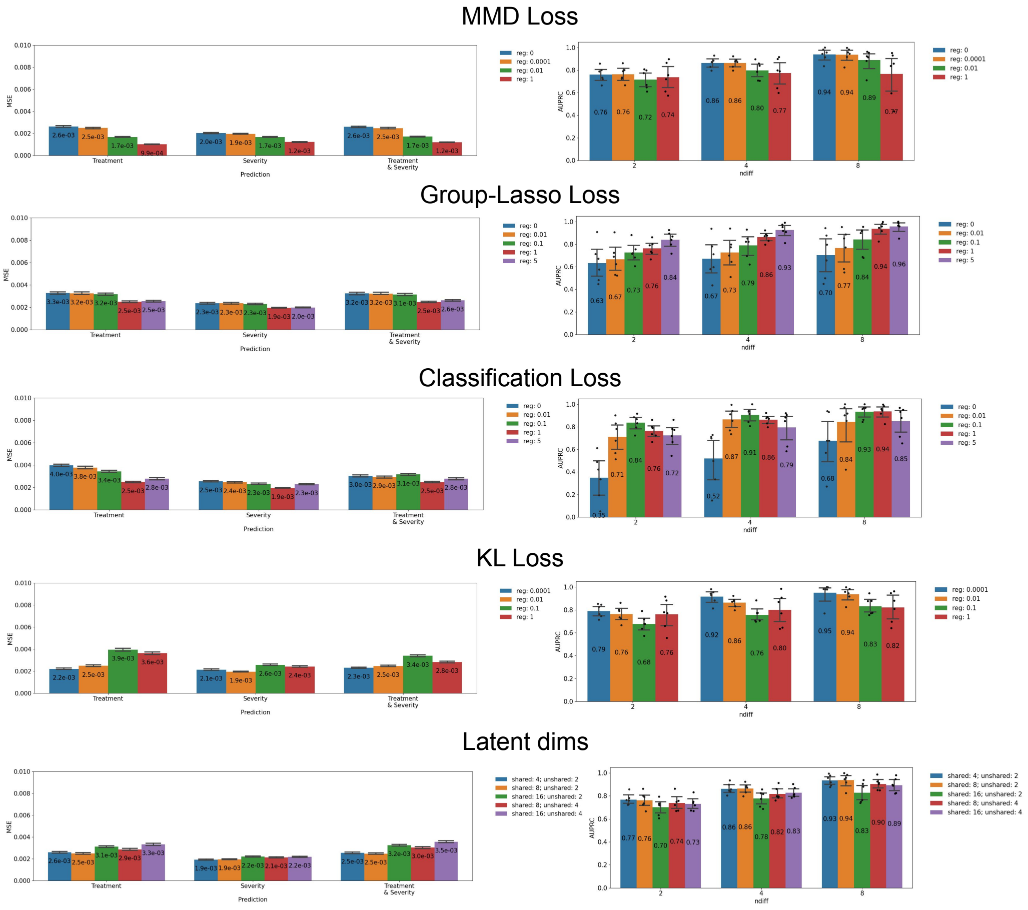

Supplementary Figure 14. Hyper-parameter sensitivity test of scDisInFact. The regularization weights of MMD loss, group-lasso loss, classification loss, KL divergence loss are tested. The latent dimensions of scDisInFact is also tested. The MSE is used to measure the perturbation prediction accuracy (left), and the AUPRC score is used to measure the CKGs detection accuracy (right). In the barplots of MSE, $n = 11214$ independent samples are included in each bar. In the barplots of AUPRC, $n = 6$ independent samples are included in each bar. The error bar represents 95% confidence interval, and the center of the error bar shows the mean. Source data are provided in the Source Data file.

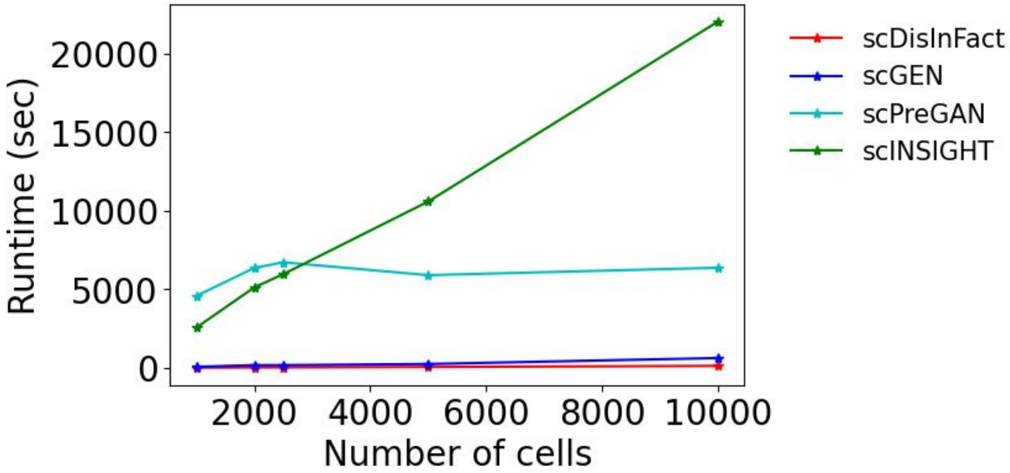

Supplementary Figure 15. The runtime comparison of scDisInFact and baseline methods including scGEN, scPreGAN, scINSIGHT on datasets of different sizes. Source data are provided in the Source Data file.

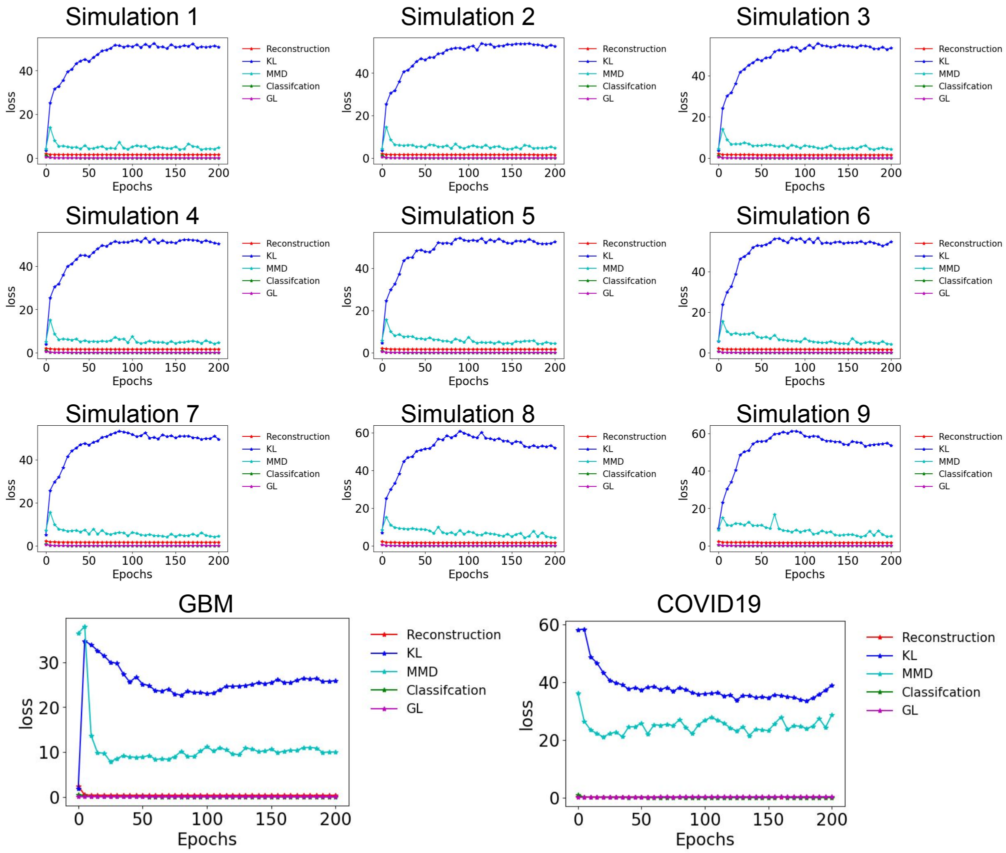

Supplementary Figure 16. Learning curves of scDisInFact on different simulated datasets (Simulation 1∼9) and real datasets (GBM, COVID19). Source data are provided in the Source Data file.

Simulation parameter setting

	Number of CKGs	Perturbation parameters
**Simulation 1**	20	2
**Simulation 2**	20	4
**Simulation 3**	20	8
**Simulation 4**	50	2
**Simulation 5**	50	4
**Simulation 6**	50	8
**Simulation 7**	100	2
**Simulation 8**	100	4
**Simulation 9**	100	8

Supplementary Table 1. The setting of simulation parameters in simulated datasets.

In-sample test (simulated dataset)

Test category	Training data (scDisInFact)	Training data (scGen/scPreGAN)	Input data	Predict data
**Condition type 1 (w/o batch effect)**	All	(ctrl & stim, severe, all batches)	(stim, severe, batch 1)	(ctrl, severe, batch 1)
**Condition type 2 (w/o batch effect)**	All	(ctrl, healthy & severe, all batches)	(ctrl, healthy, batch 1)	(ctrl, severe, batch 1)
**Condition types 1&2 (w/o batch effect)**	All	All	(stim, healthy, batch 1)	(ctrl, severe, batch 1)
**Condition type 1 (w/ batch effect)**	All	(ctrl & stim, severe, all batches)	(stim, severe, batch 2)	(ctrl, severe, batch 1)
**Condition type 2 (w/ batch effect)**	All	(ctrl, healthy & severe, all batches)	(ctrl, healthy, batch 2)	(ctrl, severe, batch 1)
**Condition types 1&2 (w/ batch effect)**	All	All	(stim, healthy, batch 2)	(ctrl, severe, batch 1)

Supplementary Table 2. The setting of training data, input data, and predicted data of scDisInFact and baseline methods in *in-sample* perturbation prediction test on simulated datasets.

Out-of-sample test (simulated dataset)

Test category	Training data (scDisInFact)	Training data (scGen/scPreGAN)	Input data	Predict data
**Condition type 1 (w/o batch effect)**	All except (ctrl, severe, all batches)	(ctrl & stim, healthy, all batches)	(stim, severe, batch 1)	(ctrl, severe, batch 1)
**Condition type 2 (w/o batch effect)**	All except (ctrl, severe, all batches)	(stim, healthy & severe, all batches)	(ctrl, healthy, batch 1)	(ctrl, severe, batch 1)
**Condition types 1&2 (w/o batch effect)**	All except (ctrl, severe, all batches)	All except (ctrl, severe, all batches)	(stim, healthy, batch 1)	(ctrl, severe, batch 1)
**Condition type 1 (w/ batch effect)**	All except (ctrl, severe, all batches)	(ctrl & stim, healthy, all batches)	(stim, severe, batch 2)	(ctrl, severe, batch 1)
**Condition type 2 (w/ batch effect)**	All except (ctrl, severe, all batches)	(stim, healthy & severe, all batches)	(ctrl, healthy, batch 2)	(ctrl, severe, batch 1)
**Condition types 1&2 (w/ batch effect)**	All except (ctrl, severe, all batches)	All except (ctrl, severe, all batches)	(stim, healthy, batch 2)	(ctrl, severe, batch 1)

Supplementary Table 3. The setting of training data, input data, and predicted data of scDisInFact and baseline methods in *out-of-sample* perturbation prediction test on simulated datasets.

Test on diversity of training data (simulated dataset)

Test category	Training data
**Hold out 1 matrix**	All except (ctrl, severe, batch 1)
**Hold out 2 matrices**	All except (ctrl, severe, batch 1), (stim, heathy, batch 2)
**Hold out 3 matrices**	All except (ctrl, severe, batch 1), (stim, heathy, batch 2), (stim, severe, batch 1)
**Hold out 4 matrices**	All except (ctrl, severe, batch 1), (stim, heathy, batch 2), (stim, severe, batch 1), (ctrl, healthy, batch 2)

Supplementary Table 4. The setting of perturbation prediction test when removing different numbers of count matrices in the training dataset.

GBM data information

Patient ID (Batch ID)	Sample ID	Condition
PW029	PW029-701	Vehicle (DMSO)
PW030	PW030-701	Vehicle (DMSO)
PW030	PW030-702	Vehicle (DMSO)
PW030	PW030-704	0.2 uM panobinostat
PW032	PW032-702	Vehicle (DMSO)
PW032	PW032-705	Vehicle (DMSO)
PW032	PW032-709	Vehicle (DMSO)
PW032	PW032-711	0.2 uM panobinostat
PW034	PW034-701	Vehicle (DMSO)
PW034	PW034-702	Vehicle (DMSO)
PW034	PW034-705	0.2 uM panobinostat
PW036	PW036-701	Vehicle (DMSO)
PW036	PW036-702	Vehicle (DMSO)
PW036	PW036-705	0.2 uM panobinostat
PW040	PW040-701	Vehicle (DMSO)
PW040	PW040-703	Vehicle (DMSO)
PW040	PW040-705	Vehicle (DMSO)
PW040	PW040-707	Vehicle (DMSO)
PW040	PW040-709	Vehicle (DMSO)
PW040	PW040-711	Vehicle (DMSO)
PW040	PW040-712	0.2 uM panobinostat

Supplementary Table 5. The patient ID, sample ID, and condition labels of samples in GBM dataset

Perturbation prediction setting (GBM dataset)

Test category	Training data	Input data	Predict data
**Treatment (w/o batch effect)**	All (except PW034-705)	(vehicle (DMSO), PW034)	(0.2 uM Panobinostat, PW034)
**Treatment (w/ batch effect)**	All (except PW034-705)	(vehicle (DMSO), PW030)	(0.2 uM Panobinostat, PW034)

Supplementary Table 6. The detailed setting of training data, input data, and predicted data in perturbation prediction test on GBM dataset.

Detailed information of in-sample test on the COVID-19 dataset

Test category	Training data (scDisInFact)	Training data (scGen/scPreGAN)	Input data	Predicted data
**Age (w/o batch effect)**	All	(moderate, 40-, all batches), (moderate, 40-65, all batches)	(moderate, 40-, Lee_2020)	(moderate, 40-65, Lee_2020)
**Disease severity (w/o batch effect)**	All	(healthy, 40-65, all batches), (moderate, 40-65, all batches)	(healthy, 40-65, Lee_2020)	(moderate, 40-65, Lee_2020)
**Age & Disease severity (w/o batch effect)**	All	(healthy, 40-65, all batches), (healthy, 65+, all batches), (moderate, 40-65, all batches)	(healthy, 65+, Lee_2020)	(moderate, 40-65, Lee_2020)
**Age (w/ batch effect)**	All	(moderate, 40-, all batches), (moderate, 40-65, all batches)	(moderate, 40-, Wilk_2020)	(moderate, 40-65, Lee_2020)
**Disease severity (w/ batch effect)**	All	(healthy, 40-65, all batches), (moderate, 40-65, all batches)	(healthy, 40-65, Wilk_2020)	(moderate, 40-65, Lee_2020)
**Age & Disease severity (w/ batch effect)**	All	(healthy, 40-65, all batches), (healthy, 65+, all batches), (moderate, 40-65, all batches)	(healthy, 65+, Arunachalam_2020)	(moderate, 40-65, Lee_2020)

Supplementary Table 7. The detailed setting of training data, input data, and predicted data of scDisInFact and baseline methods in *in-sample* perturbation prediction tests on the COVID-19 dataset.

Detailed information of out-of-sample test on the COVID-19 dataset

Test category	Training data (scDisInFact)	Training data (scGen/scPreGAN)	Input data	Predicted data
**Age (w/o batch effect)**	All except (moderate, 40-65, all batches)	(healthy, 40-, all batches), (healthy, 40-65, all batches)	(moderate, 40-, Lee_2020)	(moderate, 40-65, Lee_2020)
**Disease severity (w/o batch effect)**	All except (moderate, 40-65, all batches)	(healthy, 40-, all batches), (moderate, 40-, all batches)	(healthy, 40-65, Lee_2020)	(moderate, 40-65, Lee_2020)
**Age & Disease severity (w/o batch effect)**	All except (moderate, 40-65, all batches)	(healthy, 65+, all batches), (healthy, 40-65, all batches), (healthy, 40-, all batches), (moderate, 40-, all batches)	(healthy, 65+, Lee_2020)	(moderate, 40-65, Lee_2020)
**Age (w/ batch effect)**	All except (moderate, 40-65, all batches)	(healthy, 40-, all batches), (healthy, 40-65, all batches)	(moderate, 40-, Wilk_2020)	(moderate, 40-65, Lee_2020)
**Disease severity (w/ batch effect)**	All except (moderate, 40-65, all batches)	(healthy, 40-, all batches), (moderate, 40-, all batches)	(healthy, 40-65, Wilk_2020)	(moderate, 40-65, Lee_2020)
**Age & Disease severity (w/ batch effect)**	All except (moderate, 40-65, all batches)	(healthy, 65+, all batches), (healthy, 40-65, all batches), (healthy, 40-, all batches), (moderate, 40-, all batches)	(healthy, 65+, Arunachalam_2020)	(moderate, 40-65, Lee_2020)

Supplementary Table 8. The detailed setting of training data, input data, and predict data of scDisInFact and baseline methods in *out-of-sample* perturbation prediction tests on the COVID-19 dataset.

Neural Network parameter setting

NN	Input	Output	Activation	Dropout
**Shared Encoder-layer 1**	#genes + #batches	128	ReLU	0.2
**Shared Encoder-layer 2**	128	128	ReLU	0.2
**Shared Encoder-layer 3**	128	8	None	None
**Unshared Encoder-layer 1**	#genes	128	ReLU	0.2
**Unshared Encoder-layer 2**	128	2	None	None
**Decoder-layer 1**	8 + 2 * #condition types + #batches	128	ReLU	0.2
**Decoder-layer 2**	128	128	ReLU	0.2
**Decoder-layer 3**	128	#genes	None	None

Supplementary Table 9. The neural network parameters of scDisInFact

Supplementary Note 1: Tests on datasets with different cell type composition across batches and conditions

We conducted additional tests on scDisInFact using simulated datasets that have different cell type compositions across batches and conditions. We first tested the case where the cell type composition is different across batches in the dataset. We used the same simulated datasets in the simulation test, and randomly removed one cell type (cell type 16) or two cell types (cell types 10&16) in one data batch (batch 0). We ran scDisInFact on the processed datasets with the default hyper-parameter setting in the simulation test. We then evaluated the correctness of shared-bio factors using ARI and ASW-batch scores. Similar to the simulation test on latent space disentanglement, the ARI score is used to measure the separation of cell types, and the ASW-batch score is used to measure the alignment of cell batches. In the result (Fig. S3a), we observe that both ARI and ASW-batch scores decrease with the increase of mismatch across batches, but the model still maintains a good performance. (ARI ≥ 0.7, ASW-batch > 0.95).

We then tested the case where the cell type composition is different across conditions in the dataset. We removed all cells of cell type 16 or cell types 10&16 from the simulated data under healthy conditions, and simulated the dataset with different degrees of cell type mismatch across conditions. Again we ran scDisInFact on the processed datasets with the default hyper-parameter setting in the simulation test, and evaluated the alignment of shared-bio factors across conditions using ARI and ASW-batch scores. In the result (Fig. S3b), we also observe that both ARI and ASW-batch scores only decrease slightly with the increase of mismatch across conditions (ARI ≥ 0.7, ASW-batch > 0.95).

We performed additional tests to create the scenario of unequal cell type compositions by removing other cell types (removing one cell type: cell type 3; removing two cell types: cell types 3 and 4) and obtained similar results as those shown in Fig. S3c.

Supplementary Note 2: Tests on datasets with rare cell types

We further checked scDisInFact ability in detecting rare cell types in the dataset. We define the rare cell type as the cell type that (1) has a small cell population, and (2) does not show up in all conditions and batches. We selected one simulated dataset used in the simulation test, and picked cell type 4 as the rare cell type. When then created the rare cell type by (1) downsampling the cells of cell type 4 by 5, and (2) removing the cells of cell type 4 under the "stim" condition. We visualized the downsampled dataset with UMAP to make sure that the rare cell type cannot be identified by direct visualization. In Fig. S4, cells of cell type 4 are indeed scattered across the cell population in the visualization instead of forming a concrete cell cluster. We then ran scDisInFact on the downsampled dataset, and visualized the shared-bio factors using UMAP. The UMAP visualization on the shared-bio factors, however, shows that cells of cell type 4 is clearly separated from the other cells (Fig. S5a),

which makes it straightforward to detect the rare cell type.

Supplementary Note 3: Minimum requirements of dataset for perturbation prediction

Missing count matrices of different conditions and batches affect the performance of the model (mainly perturbation prediction) in different ways. In order for scDisInFact to make accurate perturbation prediction, we have two requirements on the dataset:

(1). The training count matrices must cover the conditions (not the condition combinations) and batches of the input and predicted count matrices. For example, when we predict the count under condition (stim, healthy, batch 0), the training data does not have to include the count matrix under (stim, healthy, batch 0), but should include at least one data matrix that has the stim condition, at least one data matrix that has the healthy condition at least one data matrix under batch 0.

(2). Enough count matrices should be provided to make sure that the effect of every condition (of different condition types) and batch can be separately learned from the data. For example, when the training data only includes (stim, healthy) and (ctrl, severe) conditions, the dataset only has the information of the joint effect of both conditions, but no information about the separate effect of each condition. Being trained on such data, the model cannot tell whether the difference of data distribution is contributed by stim/ctrl condition or healthy/severe condition. Similarly, when the training data only include (stim, batch 0) and (ctrl, batch 1), the model also cannot tell whether the difference of data distribution is contributed by stim/ctrl condition or the batch effect. To explain the requirement in a more precise format: For every two condition labels a and b of a certain condition type m, there must exist two count matrices in the dataset with all remaining condition types and batch the same but only the condition type m different (one matrix correspond to a, another matrix correspond to b).

Supplementary Note 4: The design of ablation test

We used the same simulated datasets as introduced in the simulation test (totally 9 datasets). We hold out count matrices under condition <*ctrl*, *severe*> and batches 1 & 2, and train scDisInFact on the remaining data.

For the ablation test of MMD loss, we trained two scDisInFact models with $\lambda_{mmd} = 0$ and $\lambda_{mmd} = 10^{-4}$. We then evaluated the disentanglement of the latent factors on models with and without λ_{mmd}. The disentanglement of latent factors can be further expanded into (1) removing batch effect from the shared-bio and unshared-bio factors, (2) removing condition effect from the shared-bio factors, and (3) disentangling unshared-bio factors of different condition types. To test the removal of batch effect, we measured the alignment of shared-bio and unshared-bio factors across batches. To test the removal of condition effect from the shared-bio factors, we measured the alignment of shared-bio factors across conditions. To test the disentanglement of shared-bio and unshared-bio factors, we measured the alignment of the unshared-bio factors across different conditions of the other condition types. In the three tests above, we measured the alignment of latent factors using ASW-batch

scores, and the result is shown in Figs. S12a-c.

We further evaluated the effectiveness of MMD loss in perturbation prediction. Given the input data, we used scDisInFact to predict the corresponding data under the held-out condition (<*ctrl*, *severe*>, batch 1). The prediction accuracy is measured using MSE. According to the relationship between the input and predicted condition, the perturbation prediction task can be categorized into predicting (1) treatment effect (input: <*stim*, *severe*>, batch 1), (2) severity effect (input: <*ctrl*, *healthy*>, batch 1), and (3) treatment and severity effect (input: <*stim*, *healthy*>, batch 1). The prediction accuracy is measured by MSE. We have the ground truth data for each cell (not predicting across batches, cell-level ground truth exists, see simulation procedure in Methods), the MSE is measured for each cell instead of cell type for a higher accuracy. The result is shown in Fig. S12d.

For the ablation test of classification loss, we trained two scDisInFact models with $\lambda_{ce} = 0$ and $\lambda_{ce} = 1$. We then evaluated the effectiveness of the classification loss through perturbation prediction. Again, we used scDisInFact to predict the data under the held-out condition (<*ctrl*, *severe*>, batch 1), and separated the perturbation prediction task into predicting (1) treatment effect, (2) severity effect, and (3) treatment and severity effect. The accuracy is measured with cell-level MSE, and the result is shown in Fig. S13.

For the ablation test of group-lasso loss, we trained two scDisInFact models with $\lambda_{gl} = 0$ and $\lambda_{gl} = 1$. We evaluated the effectiveness of the group-lasso loss through perturbation prediction. We used scDisInFact to predict the data under the held-out condition (<*ctrl*, *severe*>, batch 1), and measured the accuracy using cell-level MSE. The result is shown in Fig. S13.

Supplementary Note 5: The design of hyper-parameter test

We used the same simulated datasets as introduced in the simulation test (totally 9 datasets). Then we held out count matrices under condition <*ctrl*, *severe*> and batches 1 & 2, and trained scDisInFact on the remaining data. In the task of perturbation prediction, we used the fully trained model to predict the gene expression data under the held-out condition (same as the practice in the ablation test). According to the relationship between the input and predicted conditions, we separated the perturbation prediction task into predicting (1) treatment effect (input: <*stim*, *severe*>, batch 1), (2) severity effect (input: <*ctrl*, *healthy*>, batch 1), and (3) treatment and severity effect (input: <*stim*, *healthy*>, batch 1). The MSE is measured between the predicted and ground truth gene expression data of each cell (same as the ablation test). In the task of CKGs detection, we measured the detection accuracy using the AUPRC score. For each hyper-parameter that is tested, we fix the remaining hyper-parameters (using the recommended values) and only change the tested hyper-parameter:

- $\lambda_{mmd} = 0, 10^{-4}, 10^{-2}, 1$
- $\lambda_{gl} = 0, 10^{-2}, 10^{-1}, 1, 5$

- $\lambda_{ce} = 0, 10^{-2}, 10^{-1}, 1, 5$
- $\lambda_{kl}^2 = 10^{-4}, 10^{-2}, 10^{-1}, 1$
- latent dimensions (shared, unshared): $(4,2), (8,2), (16,2), (8,4), (16,4)$

Supplementary Note 6: Maximum mean discrepancy loss

Given the shared-bio factors $\mathbf{z}_s$ from two different batches and conditions (denoted separately as $\mathbf{z}_s^i$ and $\mathbf{z}_s^j$), the MMD loss can be written as

$$L_{\text{mmd}}(\mathbf{z}_s^i, \mathbf{z}_s^j, \gamma) = \mathbb{E}[K(\mathbf{z}_s^i, \mathbf{z}_s^i, \gamma)] + \mathbb{E}[K(\mathbf{z}_s^j, \mathbf{z}_s^j, \gamma)] - 2\mathbb{E}[K(\mathbf{z}_s^i, \mathbf{z}_s^j, \gamma)] \tag{1}$$

where $K(\cdot, \cdot, \gamma)$ is a Gaussian kernel function of the form $K(\mathbf{a}, \mathbf{b}) = \exp\left(-\frac{\|\mathbf{a}-\mathbf{b}\|_2^2}{2\gamma}\right)$, and γ is the hyper-parameter of the kernel function. Similar to SAUCIE[1], we calculated MMD under different γs to improve the robustness of the loss term

$$L_{mmd}(\mathbf{z}_s^i, \mathbf{z}_s^j) = \sum_{i=-6}^{6} L_{mmd}(\mathbf{z}_s^i, \mathbf{z}_s^j, \gamma = 10^i) \tag{2}$$

The same procedure also applies to unshared-bio factors $\mathbf{z}_u$.

Supplementary References

1. Amodio, M. *et al.* Exploring single-cell data with deep multitasking neural networks. *Nat. methods* **16**, 1139–1145 (2019).
