# Supplementary material for: scDisInFact: disentangled learning for integration and prediction of multi-batch multi-condition single-cell RNA-sequencing data
Source: Nat Commun. 2024 Jan 30;15:912. doi: 10.1038/s41467-024-45227-w (PMC10827746; doi:10.1038/s41467-024-45227-w)
Supplement: Supplementary file 3 — Reporting Summary [file 41467_2024_45227_MOESM3_ESM.pdf]

Corresponding author(s): Xiuwei Zhang

Last updated by author(s): Dec 13, 2023

Reporting Summary

Nature Portfolio wishes to improve the reproducibility of the work that we publish. This form provides structure for consistency and transparency in reporting. For further information on Nature Portfolio policies, see our Editorial Policies and the Editorial Policy Checklist.

Statistics

For all statistical analyses, confirm that the following items are present in the figure legend, table legend, main text, or Methods section.

n/a	Confirmed	
☐	☒	The exact sample size (*n*) for each experimental group/condition, given as a discrete number and unit of measurement
☐	☒	A statement on whether measurements were taken from distinct samples or whether the same sample was measured repeatedly
☐	☒	The statistical test(s) used AND whether they are one- or two-sided<br>*Only common tests should be described solely by name; describe more complex techniques in the Methods section.*
☒	☐	A description of all covariates tested
☐	☒	A description of any assumptions or corrections, such as tests of normality and adjustment for multiple comparisons
☐	☒	A full description of the statistical parameters including central tendency (e.g. means) or other basic estimates (e.g. regression coefficient) AND variation (e.g. standard deviation) or associated estimates of uncertainty (e.g. confidence intervals)
☐	☒	For null hypothesis testing, the test statistic (e.g. *F*, *t*, *r*) with confidence intervals, effect sizes, degrees of freedom and *P* value noted<br>*Give P values as exact values whenever suitable.*
☒	☐	For Bayesian analysis, information on the choice of priors and Markov chain Monte Carlo settings
☒	☐	For hierarchical and complex designs, identification of the appropriate level for tests and full reporting of outcomes
☐	☒	Estimates of effect sizes (e.g. Cohen's *d*, Pearson's *r*), indicating how they were calculated

Our web collection on statistics for biologists contains articles on many of the points above.

Software and code

Policy information about availability of computer code

Data collection	No software was used to collect the data.
Data analysis	scDisInFact was implemented using Pytorch (version 2.0.1+cu117) with code available at: https://github.com/ZhangLabGT/scDisInFact<br>Python packages: numpy 1.23.5, scipy 1.11.2, scikit-learn 1.3.0, umap 0.5.3, pandas 2.1.0, matplotlib 3.7.2, scGEN 2.1.0, scPreGAN commit 9a96000<br>R packages: scINSIGHT version 0.1.4, topGO version 2.48.0, SymSim 0.0.0.9000,

For manuscripts utilizing custom algorithms or software that are central to the research but not yet described in published literature, software must be made available to editors and reviewers. We strongly encourage code deposition in a community repository (e.g. GitHub). See the Nature Portfolio guidelines for submitting code & software for further information.

Data

Policy information about availability of data

All manuscripts must include a data availability statement. This statement should provide the following information, where applicable:

- Accession codes, unique identifiers, or web links for publicly available datasets
- A description of any restrictions on data availability
- For clinical datasets or third party data, please ensure that the statement adheres to our policy

All datasets used in this paper are previously published and freely available. The glioblastoma (GBM) dataset is available at Gene Expression Omnibus under

accession number GSE148842. The COVID-19 dataset is downloaded from the website with the link: https://atlas.fredhutch.org/fredhutch/covid/. The original data are also available at Gene Expression Omnibus under accession numbers GSE155673, GSE149689, and GSE150728.

Research involving human participants, their data, or biological material

Policy information about studies with human participants or human data. See also policy information about sex, gender (identity/presentation), and sexual orientation and race, ethnicity and racism.

Reporting on sex and gender	Not applicable to our study
Reporting on race, ethnicity, or other socially relevant groupings	Not applicable to our study
Population characteristics	Not applicable to our study
Recruitment	Not applicable to our study
Ethics oversight	Not applicable to our study

Note that full information on the approval of the study protocol must also be provided in the manuscript.

Field-specific reporting

Please select the one below that is the best fit for your research. If you are not sure, read the appropriate sections before making your selection.

☒ Life sciences ☐ Behavioural & social sciences ☐ Ecological, evolutionary & environmental sciences

For a reference copy of the document with all sections, see nature.com/documents/nr-reporting-summary-flat.pdf

Life sciences study design

All studies must disclose on these points even when the disclosure is negative.

Sample size	We evaluated our method by using two collections of real single-cell RNA-sequencing datasets and one collection of simulated datasets. The two real single-cell RNA-sequencing datasets are previously published and publicly available, and we used all the samples provided in the public dataset. As for the simulated dataset, we simulated 10000 cells and test our methods on all 10000 cells.
Data exclusions	No data were excluded from analysis.
Replication	With the fixed random seed, the calculation step is deterministic, and all our computation results are replicable.
Randomization	Our study does not involve allocating samples to experimental groups.
Blinding	Our study does not involve group allocation that requires blinding.

Reporting for specific materials, systems and methods

We require information from authors about some types of materials, experimental systems and methods used in many studies. Here, indicate whether each material, system or method listed is relevant to your study. If you are not sure if a list item applies to your research, read the appropriate section before selecting a response.

Materials & experimental systems

n/a	Involved in the study	
☒	☐	Antibodies
☒	☐	Eukaryotic cell lines
☒	☐	Palaeontology and archaeology
☒	☐	Animals and other organisms
☒	☐	Clinical data
☒	☐	Dual use research of concern
☒	☐	Plants

Methods

n/a	Involved in the study	
☒	☐	ChIP-seq
☒	☐	Flow cytometry
☒	☐	MRI-based neuroimaging
